# Surface modification of multilayer FePS_3_ by Ga ion irradiation

**DOI:** 10.1038/s41598-019-51714-8

**Published:** 2019-10-23

**Authors:** Heng Xu, ShangWu Wang, JianMing Ouyang, Xin He, Hao Chen, YuBo Li, Yun Liu, Rui Chen, JunBo Yang

**Affiliations:** 1 Department of Physics, National University of Defense Technology, Changsha, 410072 China; 2 College of Physics and optoelectronic engineering, Shenzhen University, Shenzhen, 518000 China; 3 Department of Mathematics, National University of Defense Technology, Changsha, 410072 China; 4 College of Physics and Electronics, Hunan University, Changsha, 410006 China; 5 Department of Biology and Chemistry, National University of Defense Technology, Changsha, 410072 China

**Keywords:** Two-dimensional materials, Chemical physics

## Abstract

In order to investigate the modification of the surface structure of FePS_3_ via Ga^+^ ion irradiation, we study the effect of thickness and Raman spectrum of multilayer FePS_3_ irradiated for 0 μs, 30 μs, and 40 μs, respectively. The results demonstrate that the intensity ratio of characteristic Raman peaks are obviously related to the thickness of FePS_3_. After Ga^+^ ion irradiation, the FePS_3_ sample gradually became thinner and the *E*_*u*_ peak and *Eg(v*_11_) peak in the Raman spectrum disappeared and the peak intensity ratio of *A*_*1g*_*(v*_2_) with respect to *A*_1*g*_*(v*_*1*_) weakened. This trend becomes more apparent while increasing irradiation time. The phenomenon is attributed to the damage of bipyramid structure of [P_2_S_6_]^4−^ units and the cleavage of the P-P bands and the P-S bands during Ga^+^ ion irradiation. The results are of great significance for improving the two-dimensional characteristics of FePS_3_ by Ga^+^ ion beam, including structural and optical properties, which pave the way of surface engineering to improve the performance of various two-dimensional layered materials via ion beam irradiation.

## Introduction

Since the successful study of graphene, various types of two-dimensional (2D) layered materials (e.g. transition metal dichalcogenides (TMDCs), black phosphorus (BPs), and hexagonal boron nitride (hBN)) have attracted enormous interests due to their unprecedented physical properties^1–4^. Recently, 2D layered transition metal phosphorus trichalcogenides (MPS_3_, M = Fe, Mn, etc.) have received enormous attentions^5–8^. As a member of MPS_3_ family, FePS_3_ is expected to be achieved by mechanical exfoliation method owning to its weak inter-layers van der Waals forces^9^. Due to its higher in-plane stiffness and lower cleavage energies than graphite, the bulk structure of FePS_3_ could be exfoliated down to the atomic thickness. Consequently, the technique of ion beam modification to achieve desired properties of various materials has been rapidly developed^10–14^. There are three effects of ion beam irradiation on 2D materials: doping effect, structural modification and defect engineering^15,16^. Generally, dopants could increase the carrier mobility while defects could decrease resistivity of materials after irradiation. Different from electron beam irradiation, ion beam irradiation has more artificially controlled conditions, such as ion species, ion energies and beam intensity^15^. In recent years, a large amount of works have focused on the changes in the properties of 2D materials through ion beam irradiation. For instance, the multilayer graphene modified low-energy (1 keV) Ar ion beam irradiation is used to study the damage and oxidation processes^16^. The insulating defect line on the monolayer graphene obtained by high-energy (30 keV) Ga^+^ ion beam corrosion is used to investigate its electrical behavior^17^. The Hall mobility of monolayer graphene also change when exposed to high-energy (35 keV) carbon ion irradiation^18^. In addition, Wang *et al*. studied the effects of surface modification of graphene induced by Ga^+^ ion beam irradiation with varying dwell times and got many meaningful results^19^. Despite the fact that there are numerous achievements and research on ion modification, the majority of the materials modified by ion beam have been focused on the graphene, and the investigate of FePS_3_ are rare. In particular, the influences of ion beam irradiation on the surface modification of FePS_3_ have not been reported yet.

Herein, we focus on investigating the surface modification of multilayer FePS_3_ via irradiating with Ga^+^ ion beam under different irradiation times. Firstly, the FePS_3_ samples are characterized by using optical microscopy, Raman spectroscopy, atomic force microscopy (AFM) and scanning electron microscope (SEM). Secondly, we study the corresponding relation between the intensity of characteristic peaks and the thicknesses of multilayer FePS_3_. Thirdly, the samples with different thicknesses were irradiated by Ga^+^ ion beam via employing focused ion beam system (FIB) with varying irradiation time, and then the changes of intensity of Raman characteristic signals are analyzed. Finally, the changes of material surface morphology after Ga^+^ ion beam irradiation are investigated by comparing the vibration modes of each peak. The results of this work are of great significance to explore the material properties of FePS_3_ by Ga^+^ ion modification.

## Results

### Preparation and characterization of FePS_3_

**Figure 1:**
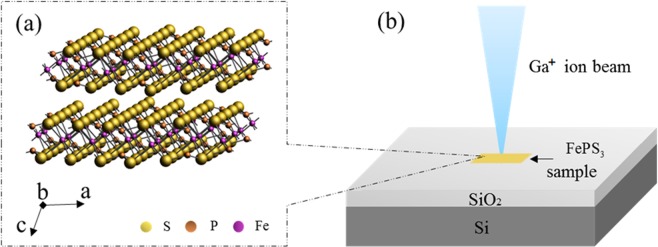
(**a**)The scheme of crystal FePS_3_ structure; (**b**) Schematic illustration of FePS_3_ irradiated by Ga^+^ ion beam.

**Figure 2:**
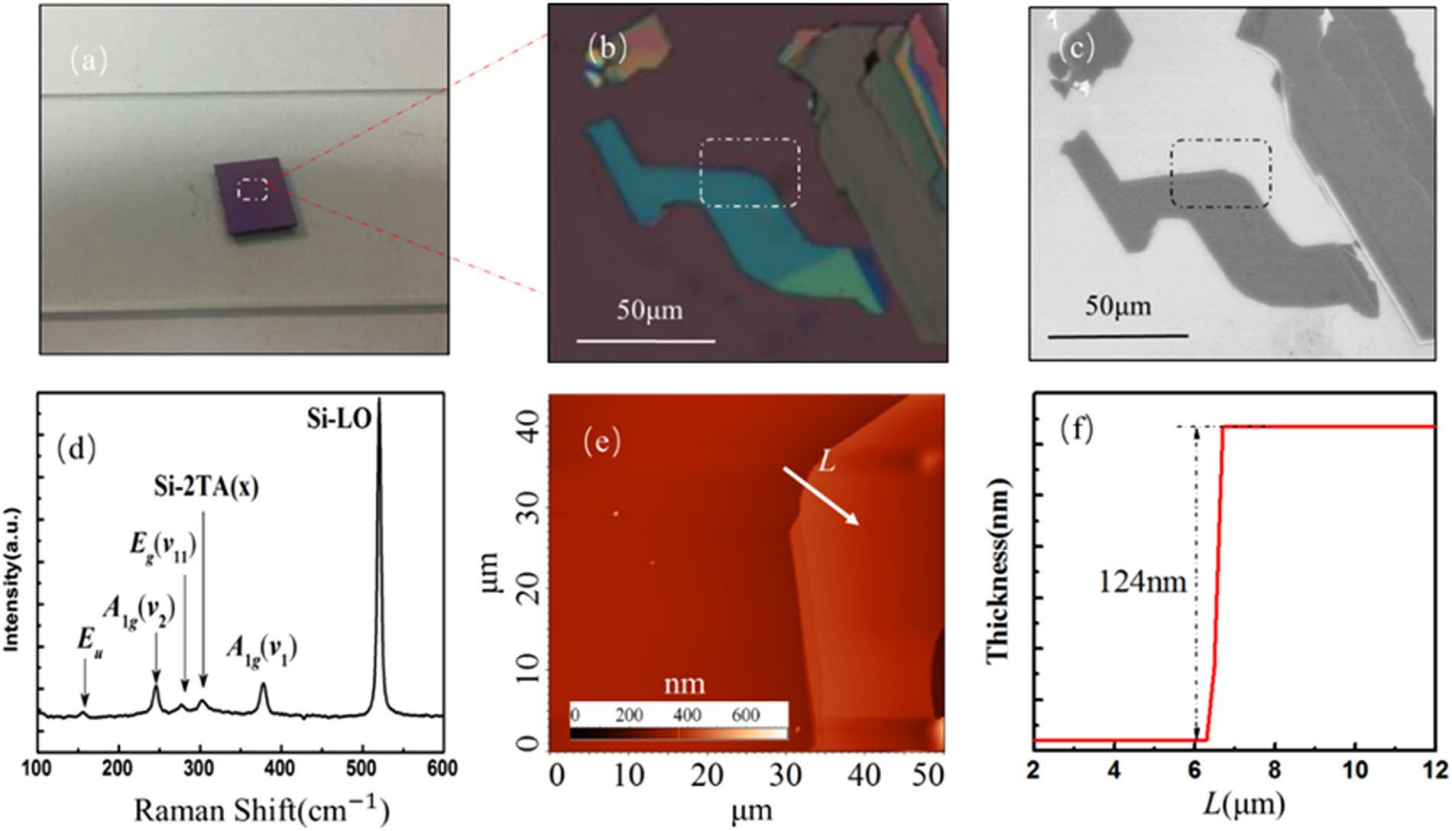
(**a**) The optical image of the FePS_3_ sample on the surface of substrate. (**b**) The optical microscope image of sample FePS_3_ prepared by mechanical exfoliation method. (**c**) The SEM image of sample. (**d**) The Raman spectrum of the FePS_3_ showing *E*_*u*_, *A*_*1g*_(*v*_2_), *E*_*g*_(*v*_11_), *Si-2TA(x)*, *A*_1*g*_(*v*_*1*_) and *Si-LO* peaks. (**e**) The AFM image of partial sample circled by white dotted line in (**b**); (**f**)The change of thickness along the white arrow line in the arrowed direction in (**e**).

The multilayer FePS_3_ samples are prepared by mechanical exfoliation from bulk FePS_3_ and then transferred onto the Si substrate with 300 nm thick SiO_2_. As shown in Fig. 1(a), FePS_3_ crystal has the CdCl_2_ type-structure, in which Fe atoms are coordinated with six S atoms, P atoms are bonded to three S atoms, and Fe atoms and S atoms are not connected by any bonds^20^. Two P atoms and six S atoms form [P_2_S_6_]^4−^ units of a bipyramid shape. Fe atoms and P atoms are sandwiched between S atoms layers to form the layer-shaped FePS_3_ monoclinic symmetrical C2/m structure^9^. The lattice parameters are that a = 5.947 Å, b = 10.300 Å, c = 6.7222 Å, and β = 107.16°^21^. Optical microscope is used to identify the FePS_3_ materials. Figure 2(a–f) show the optical image, SEM image, Raman spectra, AFM image and the thickness of sample, respectively. Raman spectroscopy is one of the most used non-destructive characterization techniques to study the properties of 2D materials. We utilize the Raman spectroscopy at room temperature with laser excitation wavelength of 785 nm. The Raman vibration modes of FePS_3_ arise from two parts of the crystal structure, corresponding to the Fe atoms and the [P_2_S_6_]^4−^ units^22^. The peaks of FePS_3_ in Raman spectrum are attributed to vibration of [P_2_S_6_]^4−^ units at room temperature. There exist three in-plane modes, *E*_*g*_-type modes (depolarized, *E*_*g*_*(v*_11_–*v*_13_)), and three out-of-plane modes, A_1g_-type modes (polarized, *A*_1*g*_*(v*_*1*_–*v*_3_)), from the vibrations of the [P_2_S_6_]^4−^ units, respectively^23^. When the FePS_3_ samples are multilayers and the [P_2_S_6_]^4−^ unit cell is doubled along the c axis, the out-of-plane vibration of two [P_2_S_6_]^4−^ units in contiguous layers becomes Raman active and the *E*_*u*_-type mode appears^23^. In general, the high-frequency peaks are mostly attributed to the molecular-like vibrations from [P_2_S_6_]^4−^ bipyramid structures, while the low-frequency modes are put down to vibrations including Fe atoms. Considering lower energy of laser wavelength of 785 nm, only *E*_*u*_ peak at ~155 cm^−1^, *E*_*g*_*(v*_11_) peak at ~277 cm^−1^, *A*_1*g*_(*v*_*1*_) peak at ~367 cm^−1^ and *A*_*1g*_*(v*_2_) peak at ~246 cm^−1^ are measured in the Raman spectrum. Other Raman active modes could be obtained by using different excitation wavelengths (such as 488 nm and 514 nm). In our experiment, the Ga^+^ ion beam irradiation is conducted using a FIB system with a kinetic energy of 30 keV and a beam current of 40 pA (at a fluence of ~2 × 10^15^ cm^−2^). The size of the irradiated areas is about 10 μm × 10 μm. Before irradiation process, FePS_3_ samples with different thickness are characterized using Raman spectroscopy and AFM. The AFM measurement are performed in the tapping mode of the scanning probe system to avoid additional damage for samples. Then the samples are irradiated by Ga^+^ ion beam for 30 μs and 40 μs, respectively.

## Experimental Results

**Figure 3:**
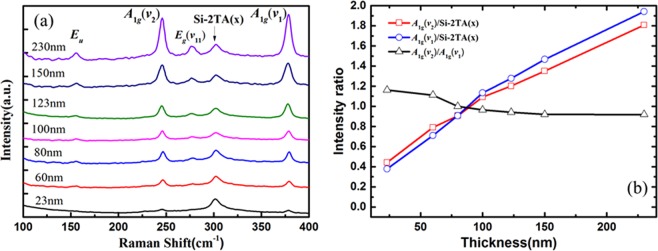
(**a**) The Raman spectrum of FePS_3_ with different thicknesses. (**b**) The change of intensity ratio of characteristic peaks with increasing thickness.

**Figure 4:**
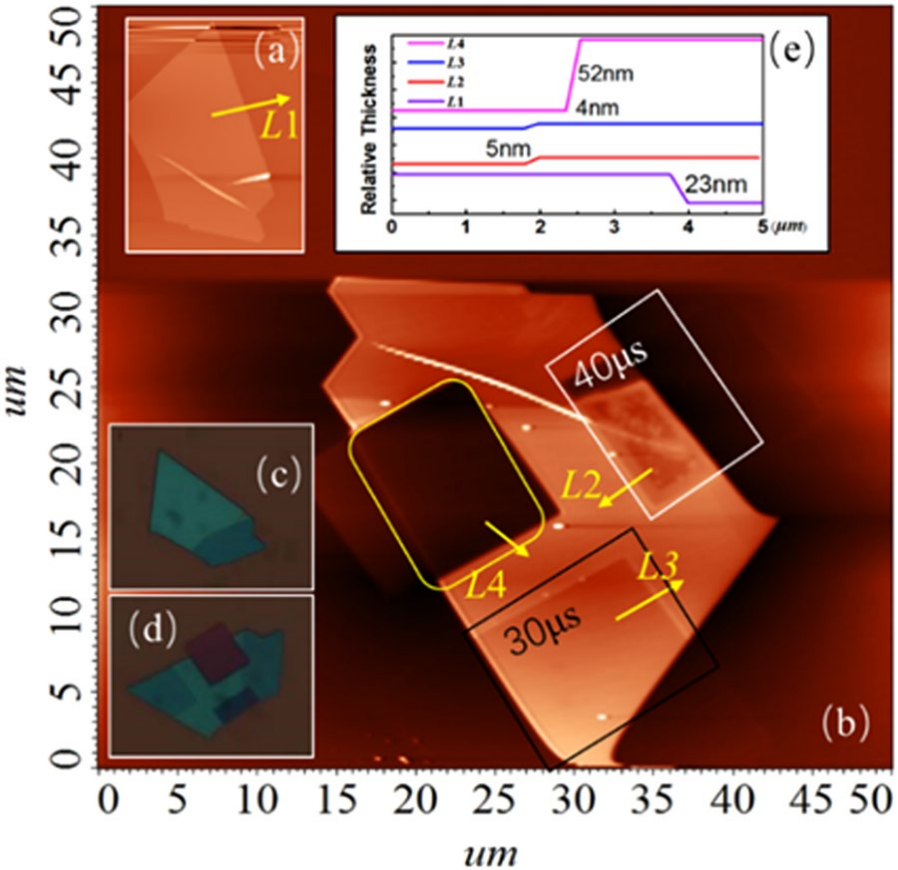
The AFM and optical images of FePS_3_ sample with thicknesses of 23 nm (S1): (**a**) The AFM image of sample S1 before irradiation. (**b**) The AFM image of sample S1 after irradiation. (**c**) The optical image of sample S1 before irradiation. (**d**) The optical image of sample S1 after irradiation. **(e**) The change of thickness along the yellow arrow line in the direction of the arrow.

**Figure 5:**
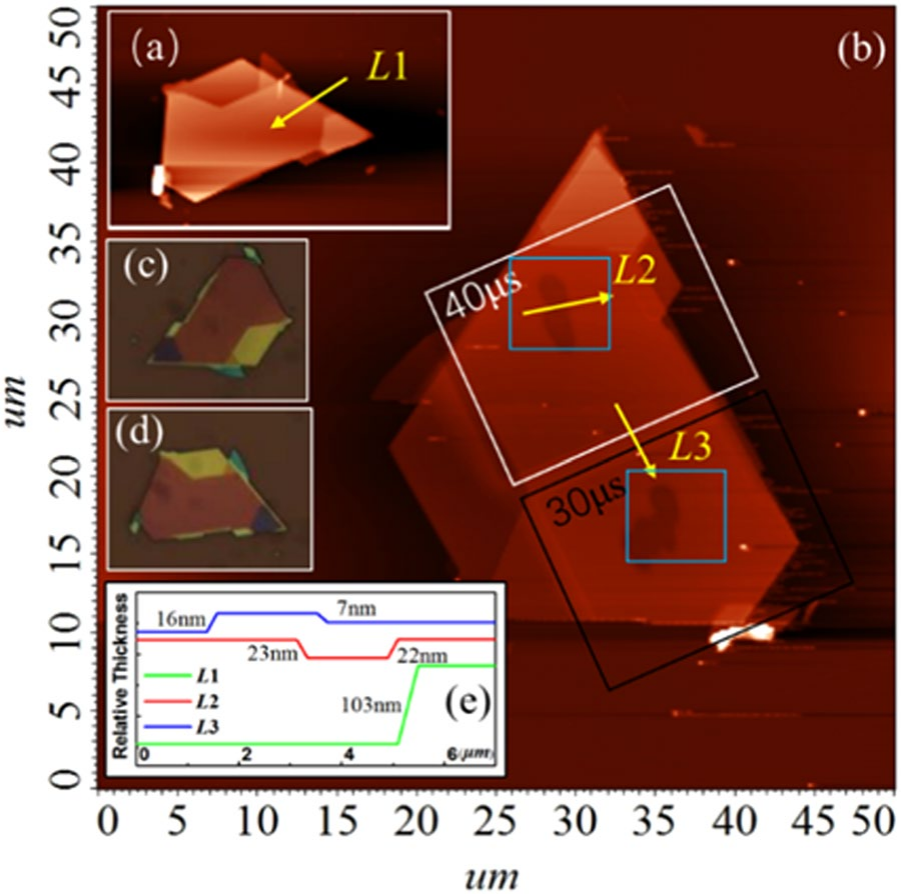
The AFM and optical images of FePS_3_ sample with thicknesses of 103 nm (S2): (**a**) The AFM image of sample S2 before irradiation. (**b**) The AFM image of sample S2 after irradiation. (**c**,**d**) The optical image of sample S2 before and after irradiation. (**e**) The change of thickness along the yellow arrow line in the direction of the arrow.

FePS_3_ samples with different thicknesses are obtained by mechanical exfoliation method. Figure 3(a) is the Raman spectra of samples, which shows that the relative peak intensity of all material characteristic peaks increases with the thickness of samples increasing in the normalized Raman spectrum. The *E*_*u*_ peak and the *E*_*g*_(*v*_11_) peak disappear when the thickness of the material is about 23 nm and the *A*_1*g*_(*v*_*1*_) peak and *A*_*1g*_(*v*_2_) peak exist though the intensity is weak. When the thickness of the sample is near 100 nm, the intensity of the *A*_1*g*_(*v*_*1*_) peak is the same as the *A*_*1g*_(*v*_2_) peak, indicating that the Raman activity of the two vibration modes are at a same level. When the thickness is increased to 230 nm, the intensity of the *E*_*g*_(*v*_11_) peak is equal to that of the substrate material *Si-2TA(x)* at ~302 cm^−1^. In order to establish the corresponding relationship of the relative intensity of each peak and the intensity ratio of the peak as a function of thickness, we select the peak intensity values of the *A*_1*g*_(*v*_*1*_), *A*_*1g*_(*v*_2_) and *Si-*2*TA(x)* peaks as the main parameters to investigate the effect of Ga^+^ irradiation with different irradiation times. As shown in Fig. 3(b), the curve is the intensity ratio versus the thickness of samples. When decreasing the thickness of samples, the intensity of the out of plane modes is increased. The intensity ratio of *A*_1*g*_(*v*_2_) with respect to *Si-2TA(x)* peaks maintains a sustained and rapid upward trend with thickness, the value of which reaches to 1.807 at 230 nm. Besides this, the intensity ratio of *A*_1*g*_(*v*_*1*_) with respect to *Si-*2*TA(x)* peaks also keeps going up and increases from 0.379 at ~25 nm to 1.812 at ~225 nm. We find that the intensity ratio of *A*_1*g*_(*v*_2_) with respect to *A*_*1g*_(*v*_*1*_) peaks decline with thickness, from 1.162 at 25 nm to 0.953 at 100 nm. And then the intensity ratio remains declining gradually as the thickness increases from 100 nm. When the thickness is 230 nm, the intensity ratio is approximately 0.918. It could be understood that the out of plane vibration modes on the contribution of Raman peaks tend to be unchanged as the thickness of sample is increasing. In addition, when the thickness is below 80 nm, the intensity ratio of the *A*_*1g*_(*v*_*2*_) peak to the *Si-2TA(x)* peak is greater than the intensity ratio of the *A*_*1g*_(*v*_*1*_) peak to the *Si-2TA(x)* peak. As the thickness increases, the values of *E*_*u*_, *A*_*1g*_(*v*_*2*_), *E*_*g*_(*v*_11_), and *A*_1*g*_(*v*_1_) with respect to Si-LO peaks remain increasing without exception. These results demonstrate that the Raman spectra of the FePS_3_ samples with different thickness have diverse characteristics, which apply a platform to study the characteristics of FePS_3_ during Ga^+^ ion irradiation by analyzing the Raman spectra. Figure 4(a,b) is the AFM image of sample S1 before and after Ga^+^ irradiation, which demonstrates that the thickness of sample S1 is obviously thinned. That could also be observed in its optical images (shown in Fig. 4(c,d)). The height profile along line in Fig. 4(e) suggests that the lateral distance of obtained sample S1 is measured to be ~23 nm. After irradiation, the thickness of S1 is measured to be dropped by 4 nm with 30 μs irradiation and 5 nm with 40 μs irradiation, respectively. Figure 5 is the AFM and optical images of sample S2 before and after Ga^+^ irradiation. The thickness of sample S2 is ~103 nm shown in Fig. 5(e). As shown in Fig. 5(a,b), the thickness of S2 is also thinned after Ga^+^ irradiation (~16 nm for 30 μs irradiation and ~22 nm for 40 μs irradiation). The optical images of S2 before and after Ga^+^ irradiation is shown in Fig. 5(c,d). Note that we find the rate of radiation-induced thinning is a little bit of change for FePS_3_ samples with different thicknesses. Considering the FIB equipment used in our experiment, it could be understood as follows: (1) due to the FePS_3_ sample with different thickness is obtained by using mechanical exfoliation method. The effective area of FePS_3_ with desired thickness is different. Generally, the thicker sample has a large effective area while thinner sample owns a smaller effective area. When using Ga^+^ ions beam to irradiate the surface of FePS_3_ samples, because it is difficult to place the material in the center of the Ga^+^ ions beam spot, which affects the rate of radiation-induced thinning. (2) Since the Ga^+^ ions beam has a fast irradiation rate, as for thinner samples, the precise focus under Ga^+^ ions imaging is hard. Therefore, there has a tradeoff between focus level and thinning rate. As a result, the rate of radiation-induced thinning for different samples might be a little different.

## Discussion

**Figure 6:**
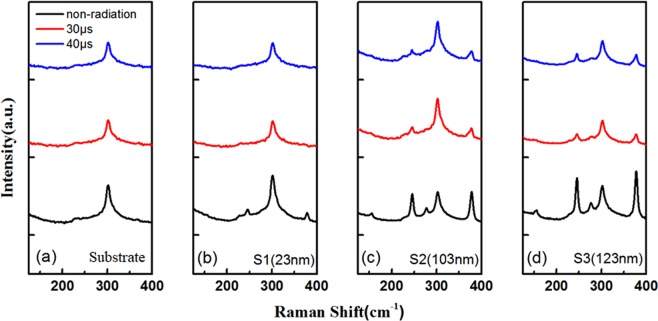
The Raman spectrum of FePS_3_ samples before and after Ga^+^ ion irradiation. (**a**) substrate; (**b**) S1; (**c**) S2; (**d**) S3.

Figure 6(a–d) are the Raman spectra of the substrate, sample S1, S2 and S3 (123 nm) before and after the Ga + ion irradiation, respectively. Due to the thin thickness of S1, the Eu peak and the Eg(v_11_) peak are not present in the Raman spectrum before irradiation, and all the characteristic peaks disappear in the normalized Raman spectra after irradiation. For sample S2, the E_u_ peak corresponding to the out-of-plane vibration of two [P_2_S_6_]^4−^ units in adjacent layers and the Eg(v_11_) peak corresponding to tangential vibration of the P-P bands and in-plane vibration of [P_2_S_6_]^4−^ unit disappeared in the Raman spectrum after Ga + ion irradiation. The intensity of A_1g_(v_2_) and A_1g_(v_1_) peaks corresponding to stretching vibration of the P-P bands and out-of-plane vibration of two [P_2_S_6_]^4−^ unit weakened to some extent. Table 1 shows the intensity ratio of characteristic peaks of sample S2 and S3. For sample S2, when the irradiation time is 30 μs, the intensity ratio of the A_1g_ (v_2_) peak with respect to the Si-2TA(x) peak is 0.520, which is 0.438 lower than that of the non-irradiated sample. The intensity ratio of the *A*_*1g*_ (*v*_*1*_) peak with respect to the *Si-*2*TA(x*) peak is 0.507, which is 0.498 lower than that of the non-irradiated sample. The intensity ratio of *A*_1*g*_ *(v*_*2*_) peak with respect to *A*_*1g*_ *(v*_*1*_*)* peak is 1.025, which is 0.072 higher than that of the non-irradiated sample. When the irradiation time is 40 μs, the value of *A*_*1g*_ *(v*_2_) peak to *Si-2TA(x)* peak, *A*_1*g*_ *(v*_*1*_) peak to *Si-*2*TA(x)* peak, and *A*_*1g*_ *(v*_*2*_) peak to *A*_1*g*_ *(v*_*1*_) peak are 0.505, 0.487, and 1.038, respectively. For sample S3, the change of intensity ratio is similar to that of S2 after irradiation. The results indicate that the intensity ratio of the *A*_1*g*_*(v*_*1*_) peak and the *Si-*2*TA(x*) peak decreases as the irradiation time increases and the intensity ratio of the *A*_*1g*_*(v*_2_) peak and the *Si-2TA(x)* peak also decreases, but the change of intensity ratio of the *A*_1*g*_*(v*_*2*_) and *A*_*1g*_*(v*_*1*_*)* peaks is opposite. The change of peaks in the Raman spectra are due to the damage of the bipyramid structure of [P_2_S_6_]^4−^ units because of the cleavage of the P-P bands and the P-S bands during Ga^+^ ion irradiation.

**Table 1:** The intensity ratio of characteristic peaks of sample S2 and S3 following different irradiation times.

Sample	Time(μs)	*A*_1g_(*v*_2_)/Si-2TA(x)	*A*_1g_(*v*_1_)/ Si-2TA(x)	*A*_1g_(*v*_2_)/*A*_1g_(*v*_1_)
S2	0	0.958	1.005	0.953
	30	0.520	0.507	1.025
	40	0.505	0.487	1.038
S3	0	1.154	1.277	0.904
	30	0.630	0.614	1.026
	40	0.626	0.598	1.046

In summary, the effect of the Ga^+^ ion beam irradiation on multilayer FePS_3_ is systematically investigated with diverse irradiation times. The corresponding relation between the thickness of samples and the intensity ratios of characteristic active peaks is obtained by analyzing Raman spectrum. The thickness of samples before and after the Ga^+^ ion beam irradiation is measured by AFM, which shows that the samples irradiated by Ga^+^ ion beam became slightly thinner. The thickness of the sample irradiated by Ga^+^ ion beams was obtained by calculating the intensity ratio of *A*_*1g*_ (*v*_2_) peak and *A*_1*g*_ (*v*_*1*_) peak, which is close to the thickness of it directly measured by AFM. Finally, compared with the un-irradiated sample with the same thickness, the *E*_*u*_ peak and *E*_*g*_(*v*_11_) peak disappeared after irradiation, indicating that the Ga^+^ ion irradiation could affect the surface structure of the sample. The peaks disappeared in the Raman spectrum are attributed to the attenuation of vibration mode due to the damage of bipyramid structure of [P_2_S_6_]^4−^ units and the cleavage of the P-P bands and the P-S bands during Ga^+^ ion irradiation. Due to the bandgap of FePS_3_ is related to the layer numbers, the material properties (such as electrical properties and nonlinear optical properties) of FePS_3_ might be engineered by Ga^+^ ions irradiation. The results are of vital importance for engineering the structural and optical characteristics of FePS_3_ by Ga^+^ ion beam irradiation.

## Methods

An optical microscopy (BX41M-LED, Olympus), a Raman spectroscopy (Senterra, Bruker), an atomic force microscopy (NT-MDT Solver SPM &SNOM) and a scanning electron microscope (HITACHI-4800) are employed to characterize the materials properties. A focused ion beam system (FIB, FEI Helios-600i) is utilized to irradiate the FePS_3_ samples with Ga^+^ ion beam.
